# When Does Da Vanci Robotic Surgical Systems Come Into Play?

**DOI:** 10.3389/fpubh.2022.828542

**Published:** 2022-01-31

**Authors:** Hao-Yun Kao, Yi-Chen Yang, Yu-Han Hung, Yenchun Jim Wu

**Affiliations:** ^1^Department of Healthcare Administration and Medical Informatics, College of Health Sciences, Kaohsiung Medical University, Kaohsiung, Taiwan; ^2^Department of Medical Research, Kaohsiung Medical University Hospital, Kaohsiung, Taiwan; ^3^Department of Nursing, Yuan's General Hospital, Kaohsiung, Taiwan; ^4^College of Humanities and Arts, National Taipei University of Education, Taipei, Taiwan; ^5^Graduate Institute of Global Business and Strategy, National Taiwan Normal University, Taipei, Taiwan

**Keywords:** robotic surgical systems, technology acceptance model, trust, intention, surgical methods

## Abstract

The emergent of medical science and technology has risen the minimally invasive surgery. Da Vinci Robotic Surgical Systems (RSS) is the trend at present. Compared with the past surgical methods, many studies related to RSS tend to explore postoperative outcomes and quality of life or compare the advantages and disadvantages than the other surgery. Few studies to understand the patients' willing who use RSS. This study mainly explores the patients' willingness and adopts the Technology Acceptance Model (TAM) as the theoretical foundation, and appended the trust concept to discuss. The study was a retrospective study and used a structured questionnaire to conduct a survey. The subjects included the patients with single-disease who had used RSS in a Medical Center of Southern Taiwan but excluded the patients with multiple disorder. This study conducted SPSS 22.0 and Smart PLS 2.0 software for statistical analysis, which included descriptive statistical analysis and applied Partial Least Squares (PLS) analysis to test the research model and to examine the established hypotheses. A total of 136 cases were collected in this study. Study validation was tested. Trust positively affects Perceived Usefulness (β = 0.550) and Perceived Ease of Use (β = 0.300). Perceived Ease of Use positively affects Perceived Usefulness (β = 0.188). Perceived Usefulness positively affects Attitude Toward Using (β = 0.589. Attitude Toward Using positively affects Behavioral Intention (β = 0.446. The relationship between perceived Ease of Use and Attitude toward Using was insignificant. Additionally, the relationship between Perceived Usefulness and Behavioral Intention was insignificant. In the research results, we found that patients are mostly in the middle and high age groups, and if the patient himself feels that RSS is extremely helpful to his illness, the intensity of his choice of intention will be high. In comparison, the information related to RSS has been clearly known, it does not directly affect the selection intention. According to age, most of the choices of RSS is based on safety and risk considerations, and it is beneficial to the patient himself, but RSS is also more expensive. We recommended that the government consider ββ reimbursing the RSS process in health insurance programs to meet the needs and expectations of patients.

## Introduction

The rapid development of medical technologies, along with improvements in medical standards and the rise of patient awareness has driven the evolution of traditional large-incision surgical practices to the development of Minimally Invasive Surgery (MIS) which minimizes incision size, blood loss and post-surgical pain (1–4). MIS practices have subsequently driven the development of Robotic Surgical Systems (RSS), which aim to improve on Conventional Laparoscopic Surgery (CLS), reduce natural tremor, and improve surgical accuracy and vision (5). These technical advantages lead to improved clinical outcomes, including reductions in postoperative complications and shortening the length of hospital stay (6).

There are very complex environments and problems in the health care system. To improve the efficiency and quality of healthcare, it often uses the development of emerging technologies and innovative medical methods, such as RSS. The current research on RSS has focused on patient safety skepticism, body dissatisfaction, and other inconsistent research results, leaving a research gap into factors that may influence patient willingness to undergo RSS (7, 8). The present study attempts to establish a prediction model for patient acceptance of RSS. Facing new medical technologies, patients usually want a voice in their medical decision-making (9–11). Previous relevant research on patient views on surgery and patient satisfaction mainly focused on the patient's perception of the degree of success of their surgery or how various surgical factors affect patient decision-making (12, 13). However, few such studies examine patient views on emerging medical technologies, especially patient decision-making and intention to adopt new surgical technologies.

Past studies have found that RSS does reduce the consumption of postsurgical medical resources, and reduces postsurgical complications, thus reducing the need for blood transfusions, respirators, and intensive care, and shortening the length of hospital stay (14, 15). The introduction of RSS also helps address the shortcomings of CLS. However, RSS is still subject to certain limitations, such as high surgery-related expenses. Seeking to increase competitiveness and avoid patient loss, hospitals are increasingly investing in RSS, taking on additional repair and maintenance costs (16, 17). This increased investment requires hospitals to maximize the use of RSS equipment, resulting in supply-induced demand and a tense relationship between physicians and patients. In addition, patients generally suffer from medical information asymmetry, leaving them unable to effectively participate in decision-making regarding their care, and thus increasing their dependence on clinicians to make critical choices (18, 19). Different patient conditions require different surgical approaches, and the choice of any particular surgical method must be carefully considered. However, the patient's lack of access to complete information leaves them little choice but to trust their physician's judgement (20, 21). More discussion is needed on the factors that patients consider when deciding to adopt RSS.

To understand these influencing factors and decision-making considerations, we apply Davis' (1989) Technology Acceptance Model (TAM) to explore the behavioral intentions of users faced with a new technology system (22, 23). This model seeks to predict and explain the factors affecting user behavior in accepting new technologies, and to explore the influence of external variables on users' personal beliefs, attitudes and intentions (24). Personal beliefs include perceived usefulness (PU) and perceived ease of use (PEOU). This study applies TAM to perform an in-depth exploration of patients' willingness to undergo RSS, coupled with the previously mentioned factors of information asymmetry and medical decision-making power asymmetry, and trust (25).

Deutsch (1962) noted that the concept of trust is at play in many fields, including the integration of science and technology acceptance models to explore the relationship between trust, PU and PEOU (26). In addition, knowledge sharing is also the key to team work (27). Trust is defined as a connected psychological state between people. In this state, people expect to cooperate with each other, giving them faith in the behavior of the other party, even in cases of uncertainty. Thus, in medical situations, the patient trusts that the medical care provider is genuinely concerned with his/her interests, and thus has the confidence necessary to accept treatment methods that may entail risk (28). In the current medical model, the physician is the key leader and decision-maker, and the relationship between physician and patient is based on mutual trust (29). The patient's trust in the doctor can be regarded as an important factor to create improved interaction and increase the patient's participation in treatment decision-making so as to jointly decide with the doctor the most suitable treatment given the patient's specific conditions (25). Based on this, this study uses “trust” as an external variable in the technology acceptance model to explore patients' willingness to choose RSS, and whether the patients' trust in physicians will affect the patients' personal beliefs, including regarding the relationship between PU and PEOU.

Therefore, based on the above research background and motivation, this study uses TAM as the theoretical basis to explore the behavioral willingness of patients to choose RSS. Retrospective research is used for data collection, and Partial Least Square (PLS) is used to verify and analyze the research model to explore the relationship and influence between the external variable trust with PU, PEOU and other aspects in TAM (30).

## Methodology

Based on the research background, motivation and related literature discussion, this study establishes a research framework to explore patients' willingness to use RSS. The research is based on the TAM proposed by Davis (1989), and considers that the RSS will be performed by the physician using the Da Vinci robotic surgical system. Therefore, the external variable Trust is added to explore the relationship between PU, PEOU, Attitude, and Intention.

This retrospective study collected data by questionnaire with Institutional Review Board (IRB) approval. The sample was collected from November 2017 to July 2018, from a medical system in southern Taiwan using RSS to treat patients for a single condition. Data was collected by structured questionnaire from patients receiving hepatobiliary, pancreatic, urological and colorectal surgery. Patients receiving treatment for multiple conditions were excluded. The final sample included a total of 136 patients. The contents of the questionnaire were explained to each patient and informed consent was secured. The questionnaire was designed based on the research purpose, past research and theoretical considerations. The questionnaire was divided into two parts, including “Patient characteristics” as the categorical variable, and “Main Questions” scored on a 5-point Likert Scale, wherein 1 = strongly disagree and 5 = strongly agree. To avoid ambiguity and unclear language in the questionnaire content which may adversely affect response validity, the researchers reviewed the questionnaire wording with two questionnaire development experts, three clinicians with RSS experience, and three teaching hospital administrators, with content validity quantified based on the Content Validity Ratio (CVR). Based on expert input, the questionnaire was modified to meet content validity standards (31, 32).

SPSS 22.0 and Smart PLS 2.03 were used for statistical analysis, including descriptive statistics and PLS. Descriptive statistics mainly analyzed the basic patient data, including gender, age, medical department, education background, economic status, occupation, prior surgical experience and prior use of Da Vinci surgery. PLS is divided into two stages: measurement model analysis and structural model analysis. Measurement model reliability and validity are assessed using the PLS algorithm, and Bootstrapping is performed to generate the path coefficient β value and *t*-value to assess statistical significance. PLS-SEM is based on regression as an analytical approach. It focuses on the explanatory power (R^2^) rather than model fit. Bootstrapping is a type of non-parental statistical inference based on resampling to makes statistical inferences when the distribution of the original population and the distribution of data sources are unknown (33).

The measurement model analysis verifies whether each theoretical measurement variable can accurately measure the potential variables, and whether there are complex measurement variables that load different potential variables, thus verifying internal consistency and construct validity. Construct Validity includes Convergent Validity and Discriminant Validity. The former refers to variables from the same dimension, which have a high degree of mutual correlation; the latter refers to variables from different dimensions, which have a low degree of mutual correlation. Structural model analysis includes analysis of the explanatory power of model fit and overall research modeling. The former tests the degree of fit between the overall research model and the observed data; the latter refers to the causal relationship between the potential variables in the model.

## Results

This study sample includes a total of 136 patients, including 114 males (83.8%) and 22 females (16.2%). Of these, 81 (59.6%) had previous surgical experience, and 14 (10.3%) had previously undergone Da Vinci robotic surgery system. Table 1 summarizes descriptive statistics for the sample as well as Table 2 shown means and standard deviations for measurement items.

**Table 1:** Descriptive analysis (*N* = 136).

**Variables**	**Contents**	***N* (%)**
Gender	Male	114 (16.2)
	Female	22 (83.8)
Age	31–49	9 (6.6)
	50–65	41 (30.1)
	66–75	58 (42.6)
	Over 75	28 (20.6)
Education	12th grade or less	55 (40.4)
	Graduated high school or equivalent	36 (26.5)
	Bachelor's degree	38 (27.9)
	Master degree or above	7 (5.1)
Medical specialist	General surgery	45 (33.1)
	Urology	86 (63.2)
	Colorectal surgery	5 (3.7)
Occupation	Medical	3 (22.0)
	Military government	32 (23.5)
	Service	91 (66.9)
	Agriculture	10 (7.4)
Disposable income (US)	1,000–2,000	89 (65.4)
	2,000–3,000	26 (19.1)
	3,000–4,000	12 (8.8)
	4,000–6,000	6 (4.4)
	Over 6,000	3 (2.2)
Operation experience	Yes	81 (59.6)
	No	55 (40.4)
Robotic surgery experience	Yes	14 (10.3)
	No	122 (89.7)

**Table 2:** Mean and standard deviation for measurement items.

**Construct dimension**	**Measurement question**	**Mean ±SD**
Trust	(Trust1) The doctor is very knowledgeable about Da Vinci surgical treatments and techniques.	4.85 ± 0.51
	(Trust2) The doctor can provide treatments that meet my needs according to my condition.	4.85 ± 0.45
	(Trust3) The doctor can fully explain to me the recovery process and postsurgical conditions.	4.85 ± 0.45
Perceive of usefulness (PU)	(PU1) Da Vinci surgery will allow me to recover quickly after surgery.	4.94 ± 0.24
	(PU2) Da Vinci surgery can relieve my pain.	4.95 ± 0.22
	(PU3) Da Vinci surgery result in a smaller and more aesthetically pleasing incision scar.	4.91 ± 0.31
	(PU41) Da Vinci surgery will reduce postoperative complications.	4.76 ± 0.55
	(PU5) Da Vinci surgery is safer than other surgical treatments.	4.82 ± 0.52
Perceive ease of use (PEOU)	(PEOU1) I clearly understand the advantages and disadvantages of Da Vinci surgery.	4.35 ± 0.81
	(PEOU2) I clearly understand the difference between Da Vinci surgery and other surgical methods.	4.46 ± 0.79
	(PEOU3) I clearly understand that Da Vinci surgery is not covered by Taiwan's National Health Insurance, and I will have to pay out of pocket.	5.00 ± 0.00
	(PEOU4) Information about Da Vinci surgery is easily accessible.	4.25 ± 0.86
	(PEOU5) I can easily ascertain the differences between Da Vinci surgery and other surgical techniques for the same condition.	4.25 ± 0.88
	(PEOU6) I have easy access to information on the advantages of Da Vinci surgery for my specific condition.	4.33 ± 0.84
Attitude (ATU)	(ATU1) Da Vinci surgery is the right choice for me.	4.85 ± 0.41
	(ATU2) Da Vinci surgery is effective.	4.82 ± 0.46
	(ATU3) Da Vinci surgery is a voluntary choice.	4.99 ± 0.12
	(ATU4) The selection of robotic surgery is under consideration.	4.62 ± 0.74
Intention (INT)	(INT1) I am willing to use Da Vinci surgery for treatment.	4.99 ± 0.12
	(INT2) If the symptoms of the disease are suitable and the doctor's skills permit, I will specify the use Da Vinci surgery.	4.76 ± 0.58
	(INT3) I think it is worthwhile to use Da Vinci surgery.	4.86 ± 0.43
	(INT4) I would recommend Da Vinci surgery to others.	4.87 ± 0.44

In the reliability and validity analysis, the composite reliability (CR) value of each dimension of this study does not fall below 0.5 and thus meets the standard. Table 3 shows the CR and Cronbach's α values of each dimension. Convergent Validity refers to whether the questionnaire items in each facet can converge based on factor loading (FL) and the average extraction variance (AVE) for each facet, where the FL and AVE values for all items should exceed 0.5. Table 4 shows the Convergent Validity analysis results. This study was conducted using the method proposed by Chin (1998), in which discriminant validity is demonstrated by the square root of AVE of each facet exceeding the correlation coefficient between the facets (34). The results are shown in Table 5.

**Table 3:** The CR and Cronbach's α for constructs.

**Constructs**	**Items**	**CR**	**Cronbach's α**
Trust	3	0.820	0.670
PU	5	0.863	0.801
PEOU	6	0.848	0.856
INT	4	0.744	0.670
ATU	4	0.759	0.688

**Table 4:** Convergent validity analysis results.

**Constructs**	**Item**	**Factor loading**	**AVE**
Trust	Trust1	0.573	0.611
	Trust2	0.885	
	Trust3	0.849	
PU	PU1	0.726	0.558
	PU2	0.800	
	PU3	0.642	
	PU4	0.758	
	PU5	0.799	
PEOU	PEOU1	0.729	0.634
	PEOU2	0.762	
	PEOU3	0.000	
	PEOU4	0.751	
	PEOU5	0.848	
	PEOU6	0.899	
ATU	ATU1	0.902	0.626
	ATU2	0.882	
	ATU3	0.510	
	ATU4	0.188	
INT	INT1	0.231	0.643
	INT2	0.524	
	INT3	0.949	
	INT4	0.885	

**Table 5:** Discriminant validity results (*N* = 136).

**Constructs**	**Trust**	**PU**	**PEOU**	**INT**	**ATU**
Trust	1.000				
PU	0.606^*^	1.000			
PEOU	0.299	0.351	1.000		
INT	0.591	0.546	0.330	1.000	
ATU	0.532	0.628	0.309	0.607	1.00

* *The root square of AVE for constructs*.

**Figure 1:**
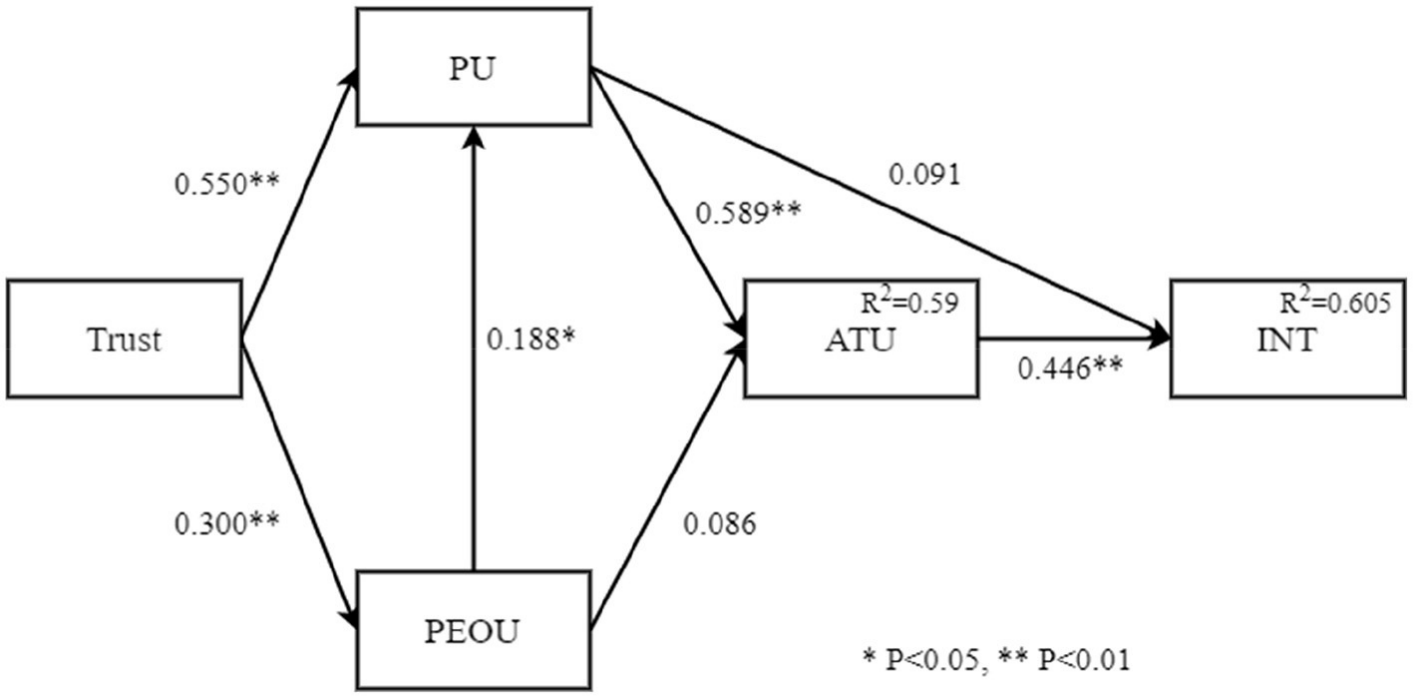
The path analysis of research model (*N* = 136).

This research establishes 5 hypotheses as follows: Trust positively affects PU (β = 0.550, T = 4.527), Trust positively affects PEOU (β = 0.300, T = 4.579), PEOU positively affects PU (β = 0.188, T = 2.242), PU positively affects ATU (β = 0.589, T = 4.835), and ATU positively affects BI (β = 0.446, T = 2.416). Two hypotheses are found to not be statistically significant and are thus not supported, namely PEOU and ATU, PU and BI. Figure 1 shows the verification of the research model path.

## Discussion

The results of this study found that the trust relationship between patients and physicians affects patients' understanding of RSS information and their decision-making regarding the procedure (35). Patients' trust in their physicians is regarded as a core factor in the relationship, allowing patients to better understand RSS and its treatment efficacy. In addition, PEOU is found to positively affect PU, which is consistent with the previous results, suggesting that patients who can easily obtain and clearly understand RSS information will feel confidence in determining its potential benefit to themselves. Unlike in previous studies, PEOU is found to not directly affect attitudes toward RSS usage. This study finds that PEOU is affected by PU. Since the definition of PU in this study was modified from Davis (1989), the expression was slightly adjusted but the meaning is consistent following modification and expert validation. The original definition refers to the information technology system used by the user himself or herself, where systems which are easy to operate will result in increased user acceptance. However, in this case, the RSS is operated by a physician. Therefore, this study revised the definition of perceptual ease of use based on the recommendations of experts in the relevant field to obtain a better measurement and interpretation of this dimension (22, 25).

In addition, the results show that, even if patients can easily obtain RSS-related information and understand its purpose, there is no positive correlation with patients' attitudes toward using RSS. PU does not directly affect the willingness to use, but it will indirectly affect the willingness to use through attitude of use. This is the same concept as PU, because RSS is operated by the physician rather than by the patient, where the trust factor provides the patient confidence in the benefits of RSS. However, the results show that the use of RSS to help patients only affects willingness to use indirectly through their attitude (24, 25). That is, in addition to providing patients with relief from their symptoms, willingness to use RSS depends on the patient having a positive attitude toward the technology and confidence that RSS is the correct choice for their condition.

To sum up, use of RSS has increased significantly in recent years and provides advantages over traditional surgery or endoscopic surgery for complicated procedures in difficult surgical sites. Since the introduction of Da Vinci robotic surgery system in Taiwan in 2004, at least 30,000 operations have been completed, and with 80% conducted in the past three years. The present research results indicate that RSS is predominantly used by middle-aged and elderly people, but application is limited by financial considerations as RSS is only covered by Taiwan's social insurance scheme for the treatment of prostate cancer. Given the treatment advantages provided by RSS, the government should consider expanding social insurance coverage to include RSS treatment for more complex lower rectal cancer, or pre-liver transplantation surgery. Continuing advances in medical technology and the prevalence of minimally invasive surgery also provide patients and doctors with additional options for suitable surgical approaches. Therefore, hospitals should seek to implement shared medical decision-making, reducing information asymmetry for patients and empowering them to actively participate in the surgical decision-making process (10, 19).

This research makes two distinct contributions. This study uses TAM to assess the behavior and intention of RSS patients, unlike other studies which primarily focus on the effectiveness of Da Vinci robotic surgery system. This research also proposes that trust will affect patients' personal beliefs in terms of their perception of the surgery as being helpful, or the availability of information about the surgery to make informed decisions. At the same time, this research also helps to explore factors affecting patient willingness to adopt advanced surgical techniques (25, 29).

In terms of practical contributions, this study is mainly based on behavioral intention, and finds that most patients have a certain degree of understanding of RSS, but their most important consideration in determining their willingness to undergo RSS is the effectiveness of the procedure. Therefore, clinicians should seek to clearly explain the benefits of RSS for the specific condition of each patient. Patients' trust in their doctors determines their willingness to accept doctors' suggestions, which in turn will affect the patients' views on the relevant medical information available to them, which serves as the foundation for further decision making. Therefore, doctors serve a key role in communicating critical medical information, and must seek to empathize with their patients, thereby preserving trust and maintaining treatment effectiveness (29, 35).

The TAM was used to measure new technologies in their use and adoption. The literature indicates that modifying the model was primarily adding or removing variables and, in some cases, adding moderators or mediators. The model has limits identified in the literature as the problem of reliably quantifying behavior in an observed survey. In addition, there are notable criticisms identified in the literature, such as the failure of the TAM to notice other issues, such as costs and structural imperatives that push users to adopt an innovation. The TAM will continue to be accepted and modified based on the successful implementation of any new health care technology. This exploratory cross-sectional study does not account for differences between various medical specialties, which should be addressed in future research designs that can also compare the selection factors and intentions of patients who have and have not previously used RSS, thus providing more representative results.

## Data Availability Statement

The raw data supporting the conclusions of this article will be made available by the authors, without undue reservation.

## Ethics Statement

The studies involving human participants were reviewed and approved by Kaohsiung Medical University. The patients/participants provided their written informed consent to participate in this study.

## Author Contributions

H-YK and YW: supervision of the project, design of the research, organization of experiment conduction, data analysis and interpretation, writing, and revision of the article. Y-HH and Y-CY: organization of experiment conduction, data analysis and interpretation, and writing of the article. All authors contributed to the article and approved the submitted version.

## Funding

The authors gratefully acknowledge the Grant Support Number: MOST 110-2511-H-037-008-, which was awarded by the Ministry of Science and Technology (MOST), Taiwan. In addition, this study was supported by Grant Support Number: KMU-M110016 from Kaohsiung Medical University, Taiwan.

## Conflict of Interest

The authors declare that the research was conducted in the absence of any commercial or financial relationships that could be construed as a potential conflict of interest.

## Publisher's Note

All claims expressed in this article are solely those of the authors and do not necessarily represent those of their affiliated organizations, or those of the publisher, the editors and the reviewers. Any product that may be evaluated in this article, or claim that may be made by its manufacturer, is not guaranteed or endorsed by the publisher.
